# Exploring Neuronal Differentiation Profiles in SH-SY5Y Cells through Magnetic Levitation Analysis

**DOI:** 10.1021/acsomega.3c08962

**Published:** 2024-03-22

**Authors:** Rumeysa Bilginer Kartal, Ahu Arslan Yildiz

**Affiliations:** Department of Bioengineering, Izmir Institute of Technology (IZTECH), 35430 Izmir, Turkey

## Abstract

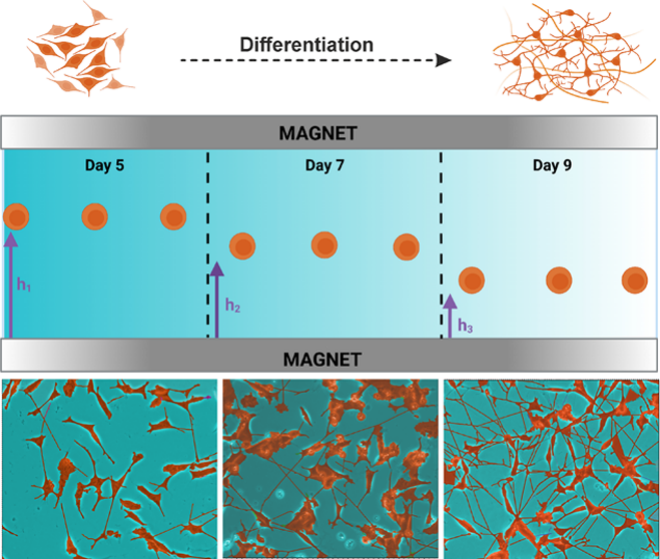

Magnetic levitation (MagLev) is a powerful and versatile technique that can sort objects based on their density differences. This paper reports the sorting of SH-SY5Y cells for neuronal differentiation by the MagLev technique. Herein, SH-SY5Y cells were differentiated with retinoic acid (RA) and brain-derived neurotrophic factor (BDNF). Neuronal differentiation was confirmed by neurite extension measurement and the immunostaining assay. Neurites reached the maximum length on day 9 after sequential treatment with RA-BDNF. Neuronal marker expression of un-/differentiated cells was investigated by β-III tubulin and neuronal nuclei (NeuN) and differentiated cells exhibited a higher fluorescence intensity compared to un-/differentiated cells. MagLev results revealed that the density of differentiated SH-SY5Y cells gradually increased from 1.04 to 1.06 g/mL, while it remained stable at 1.05 g/mL for un-/differentiated cells. These findings signified that cell density would be a potent indicator of neuronal differentiation. Overall, it was shown that MagLev methodology can provide rapid, label-free, and easy sorting to analyze the differentiation of cells at a single-cell level.

## Introduction

Differentiation of neuroblastoma cells is a critical step for neuroscience in terms of having similar morphological and biochemical properties in vivo and is prominent for neural development or^1−3^ the creation of neurodegenerative disease models for drug discovery and screening.^4,5^ The SH-SY5Y neuroblastoma cell line is widely used in neuronal studies, such as differentiation^6,7^ and neurodegenerative disease modeling.^8^ In literature, retinoic acid (RA) has been widely used to differentiate SH-SY5Y cells, and additionally, brain-derived neurotrophic factor (BDNF) has been used to increase the effect of neuronal differentiation.^4,9−14^ During cellular differentiation, biochemical and morphological changes occur, such as extension in neurites, alterations in neuronal marker expression levels and cell density, and accumulation of some neurotransmitters, thus becoming phenotypically similar to primary neurons.^13,15−18^ Up to now, neuronal differentiation has been monitored and detected by conventional methods; while neurite extension has been observed by light microscopy, neuronal marker expression has been detected by flow cytometry, immunostaining, quantitative polymerase chain reaction (qPCR), and Western blot.^11,15−17,19,20^ Although these methods are effective, they are time-consuming, expensive, and labor intensive; therefore, alternative methodologies are required.

Magnetic levitation (MagLev) is a newly developed simple and cost-effective methodology that can perform sensitive density-based measurements without the need for labeling or tags.^21−24^ MagLev can be applied to biological and nonbiological samples, since it provides sensitive measurements in density-based separation.^25−28^ Recently, this method has been utilized to separate cells with different characteristics based on their density differences; for instance, healthy and cancerous cells.^21^ Moreover, healthy cells from anemia cells could be separated easily based on their density via MagLev platform, which was integrated with a smartphone without the need for a microscope, and this system was able to give results in less than 15 min.^29^ In another study, MagLev was applied to separate *Escherichia coli* and *Saccharomyces cerevisiae* based on density differences with ∼100% efficiency.^30^ Hence, MagLev is a good candidate to investigate the neuronal differentiation of cells based on their density, because cell density changes occur during differentiation, and it is possible to distinguish populations of cells that are un-/differentiated based on their density.^25,31,32^

MagLev offers a promising way to investigate neuronal differentiation based on cell density. During cellular differentiation, significant biochemical and morphological changes occur. Cell density is one of the properties that can be affected by the differentiation process.^32,33^ In a reported case, the cell density was changed based on the differentiation status during cortical development;^32^ in another case, it was observed that cell density had a significant role in the differentiation pathway.^33^ Therefore, cell density can be a cue to discriminate un-/differentiated cells using the MagLev method. The application of Maglev in this context has significant implications, as it demonstrates simple and rapid detection of neuronal differentiation at a single-cell level. Besides, sorting of the cells based on their density can obviate the drawbacks of conventional techniques such as being time-consuming, labor intensive, and need for tags. Considering all this, the study aims to sort un-/differentiated cells based on their density differences via MagLev, which will allow the opening of new avenues for further advancements to provide insights into neuronal differentiation from different perspectives, contributing to cell biology research. For this purpose, SH-SY5Y cells were differentiated with RA and BDNF through 9 days, and neurite extension of un-/differentiated cells was measured for verifying differentiation, first. Later, immunostaining of β-III tubulin and neuronal nuclei (NeuN) biomarkers was carried out for investigating neuronal differentiation, followed by fluorescence intensity calculation. After characterization, un-/differentiated cells were sorted via MagLev, where Gadobutrol (Gx) was utilized as a paramagnetic agent. Prior to cell sorting, the cytotoxicity of Gx was examined by live/dead and MTT assays. Afterward, cells were levitated in the MagLev platform based on their density. This study signifies that Maglev methodology allows rapid and simple detection of neuronal differentiation at the single-cell level without the use of labeling.

## Results and Discussion

### Morphology and Neurite Extension Analysis of Un-/Differentiated SH-SY5Y Cells

**Figure 1:**
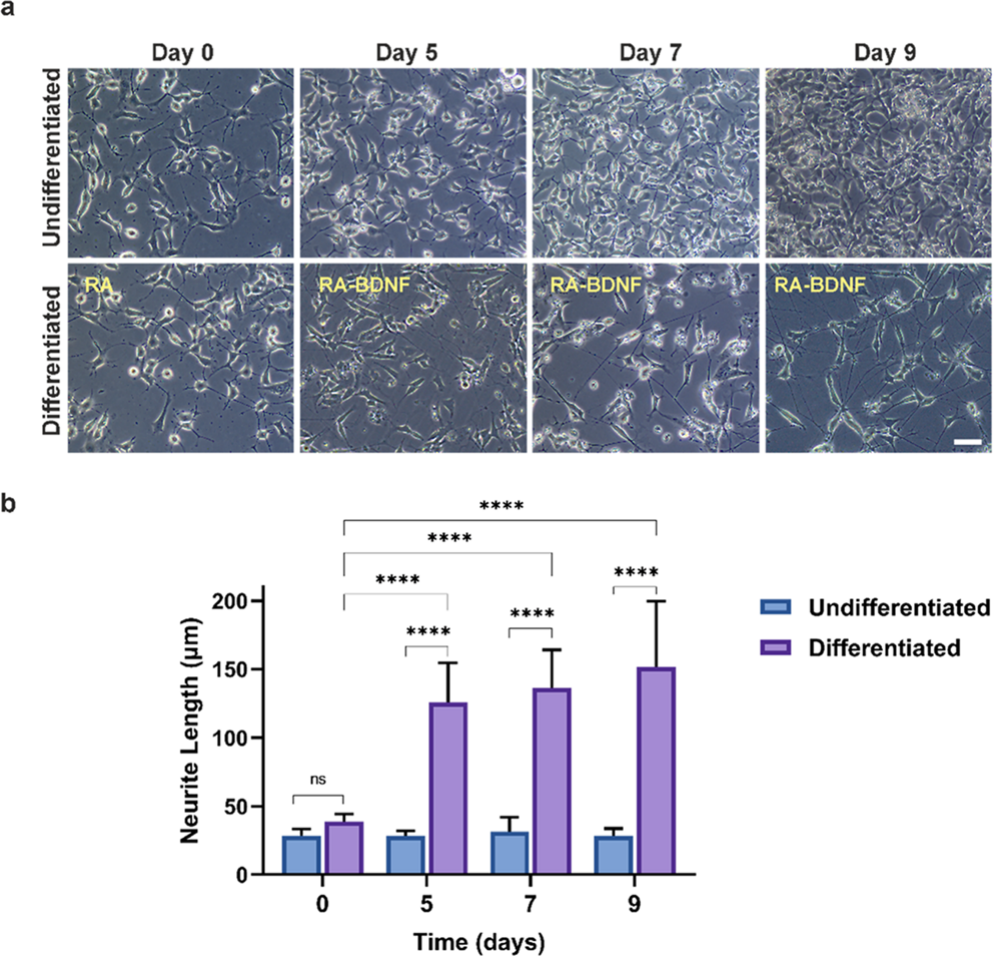
(a) Morphology and (b) neurite extension analysis of un-/differentiated SH-SY5Y after sequential treatment by RA and RA-BDNF for 9 days (*n* = 10, ns: not significant, ****p < 0.0001). Scale bar: 50 μm.

Differentiation of neuroblastoma cells is a complex process that is governed by several factors that also include neurotrophins. RA induces tyrosine kinase receptor B (TrkB) expression, which is necessary for binding of BDNF.^34,35^ After that, BDNF activates phosphatidylinositol 3-kinase (PI 3-K) and extracellular regulated kinase (ERK) pathways, which play a role in cell survival and neuritogenesis.^13,36−38^ After addition of RA, neurite extension was observed from day 0 to day 5 (Figure 1a). On day 5, BDNF was supplemented to enhance the effect of RA, and it provided longer and more branched neurites, especially on day 9. Starting from day 5, cells began to exhibit the neuronal phenotype, confirming that RA and BDNF induced differentiation of SH-SY5Y cells. Neurite lengths were measured, and extensions were calculated by Neuron J software. Figure 1b shows the comparison of neurite lengths between un-/differentiated SH-SY5Y calculated from day 0 to day 9. The neurite length of un-/differentiated cells ranged between 28 and 31 μm, whereas that of differentiated ones reached up to 150 μm. When only RA was used, the neurite extension was temporary, and after day 5, neurites started to shorten (Supporting Figure 1). BDNF has a synergetic effect with RA, which leads to an extension of neurite length reaching 150 μm from 125 μm, permanently. Sequential treatment of RA and RA-BDNF resulted in almost 4-fold increase of neurite lengths on day 9 compared to day 0, which shows the differentiation capability of a combined approach. This result is in concordance with the conclusion of another report in which was observed that the neurite length was shorter when SH-SY5Y cells were only treated with RA, compared to RA-BDNF.^17^ In other studies, different outcomes were reported. In one case prolonged RA treatment of more than 5 days could not maintain a homogeneous population of SH-SY5Y, leading to an increment of S-type cells, and the addition of BDNF provided more branched and abundant neurites,^13^ while in another report, RA treatment exceeding 3 days increased the apoptotic cell death percentage.^39^ These findings collectively elicit that RA treatment initiates the differentiation process, but it does not differentiate cells efficiently when it is used solely, underscoring that a combined approach achieves efficient neuronal differentiation.

### Investigation of Neuronal Differentiation by Immunostaining

**Figure 2:**
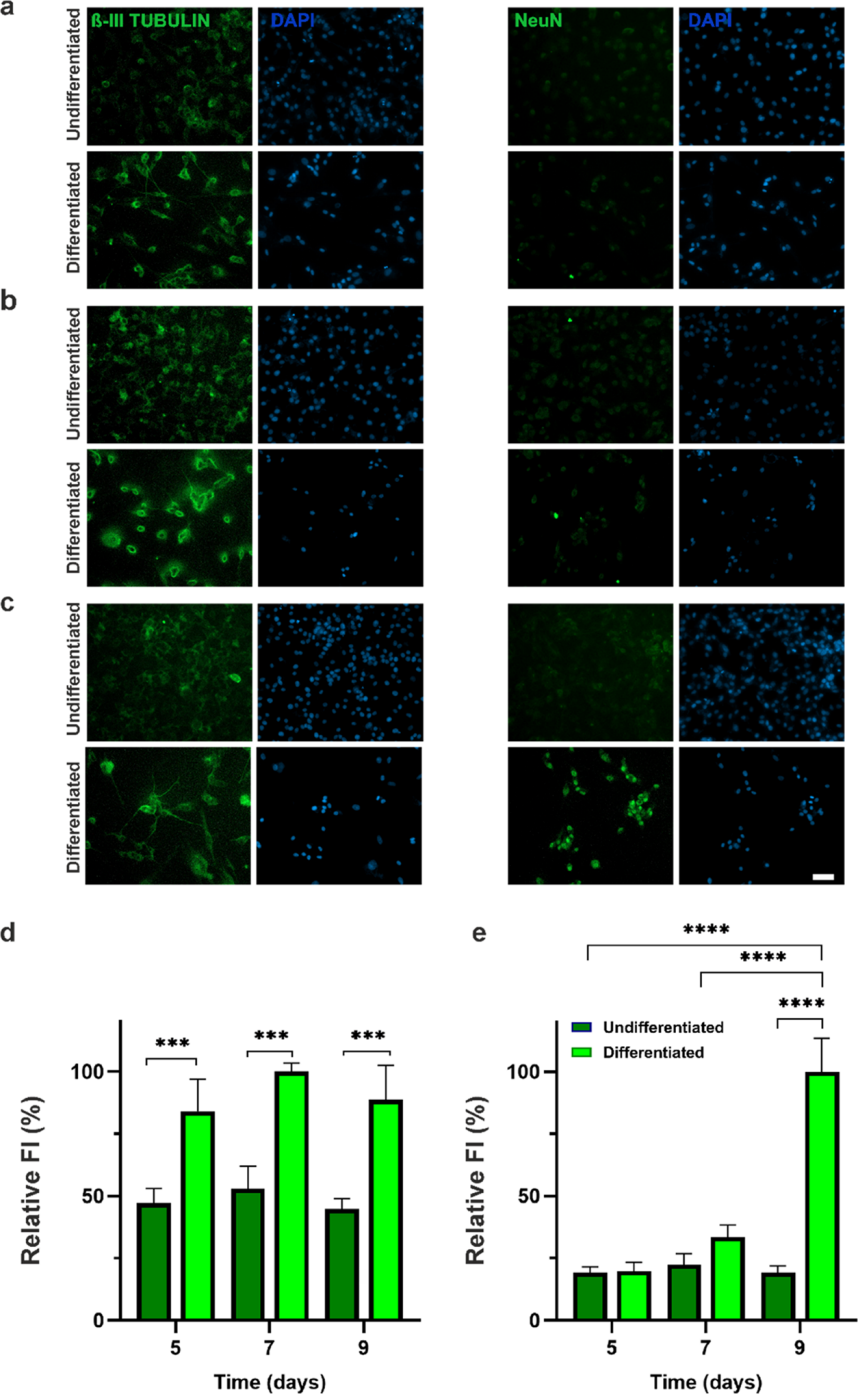
Immunostaining of β-III tubulin and NeuN for un-/differentiated SH-SY5Y cells on (a) day 5, (b) day 7, and (c) day 9 (blue: DAPI, green: β-III tubulin and NeuN). Scale bar: 50 μm. Relative fluorescence intensity (FI) of (d) β-III tubulin and (e) NeuN (*n* = 6, ns: not significant, ***p < 0.001, ****p < 0.0001).

β-III Tubulin is a neuron-specific class of tubulin that increases with the rate of neuronal differentiation. Neuronal nuclei (NeuN) is only observed when cells are differentiated.^40,41^ Therefore, neuronal differentiation of SH-SY5Y was confirmed via β-III tubulin and NeuN immunostaining. Figure 2 demonstrates β-III tubulin and NeuN expression of un-/differentiated cells. β-III tubulin was expressed slightly on un-/differentiated cells and clearly observed in differentiated groups (Figure 2a–c). The relative fluorescence intensity of β-III tubulin was observed to be 2-fold higher for all time intervals in differentiated cells compared to the un-/differentiated control groups (Figure 2d). NeuN was weakly expressed in un-/differentiated groups (Figure 2a–c), and the relative fluorescence intensity of NeuN in differentiated cells increased 5-fold on day 9 compared to the un-/differentiated control groups (Figure 2e). In the literature, increase of β-III tubulin and NeuN expression were observed after the differentiation of SH-SY5Y, which supports our findings.^17,42^

### Assessment of Gadobutrol Cytotoxicity

**Figure 3:**
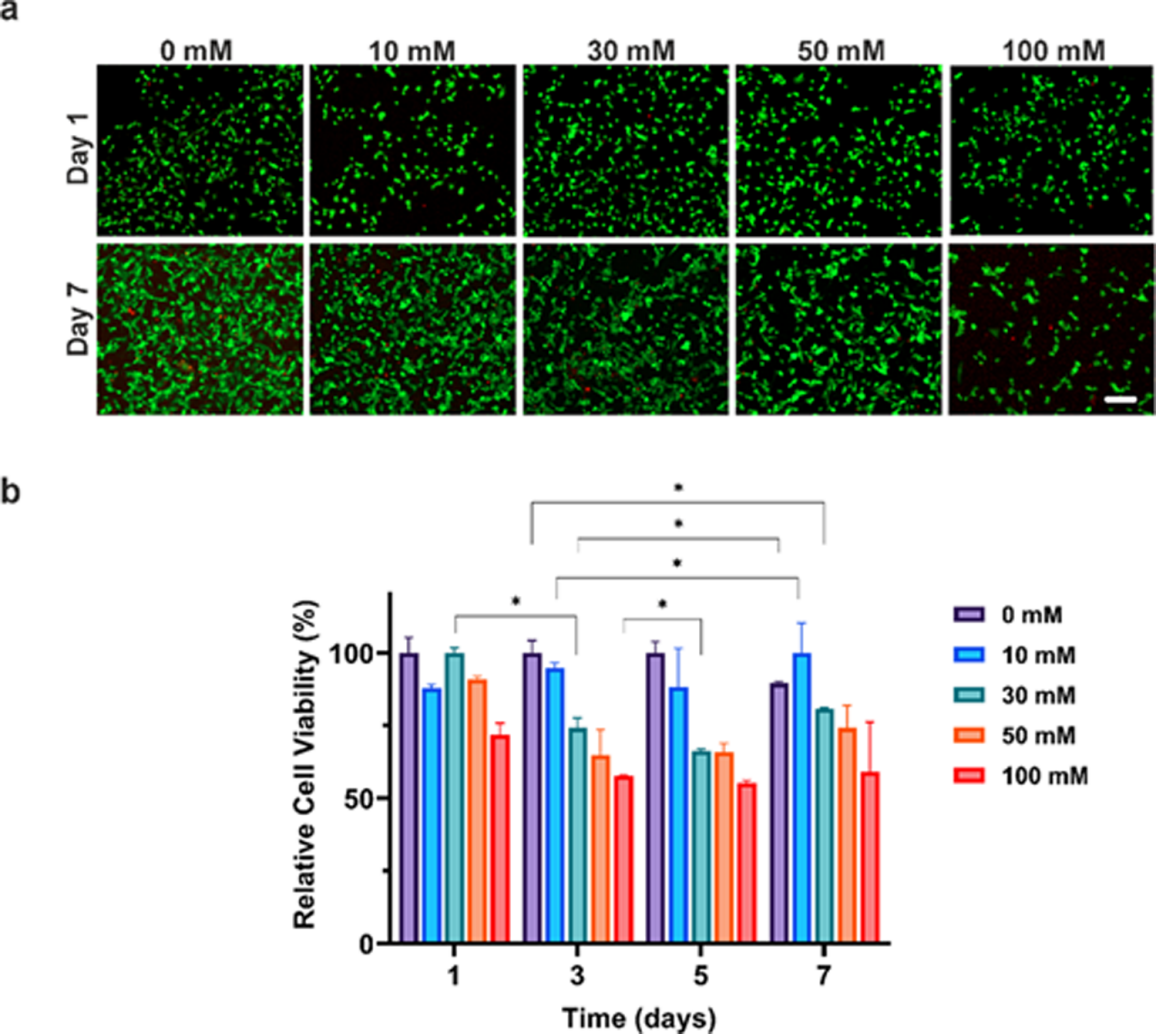
Cytotoxicity assessment of 10, 30, 50, and 100 mM Gx on SH-SY5Y cells by (a) live/dead assay on day 1 and day 7 (scale bar: 100 μm) (green: live cells, red: dead cells) and (b) MTT assay for 7-day culture (*n* = 3, *p < 0.05).

To sort the cells via MagLev, Gx was used as a paramagnetic agent due to its low cytotoxicity and high separation capability.^22,43^ Evaluation of Gx cytotoxicity on the SH-SY5Y cell line was carried out by live/dead and MTT assays. Figure 3a shows that Gx exhibited the highest cell .viability in the concentration range of 10–30 mM. For all Gx concentrations, even for 100 mM, cell viability was above 50% according to the MTT results on day 7, which was also consistent with the live/dead findings (Figure 3). The low cytotoxicity profile of Gx can be attributed to the slow dissociation of gadolinium ions (Gd^3+^) in Gx compared to different gadolinium-based paramagnetic agents.^43^

### Sorting of Un-/Differentiated Cells via MagLev

**Figure 4:**
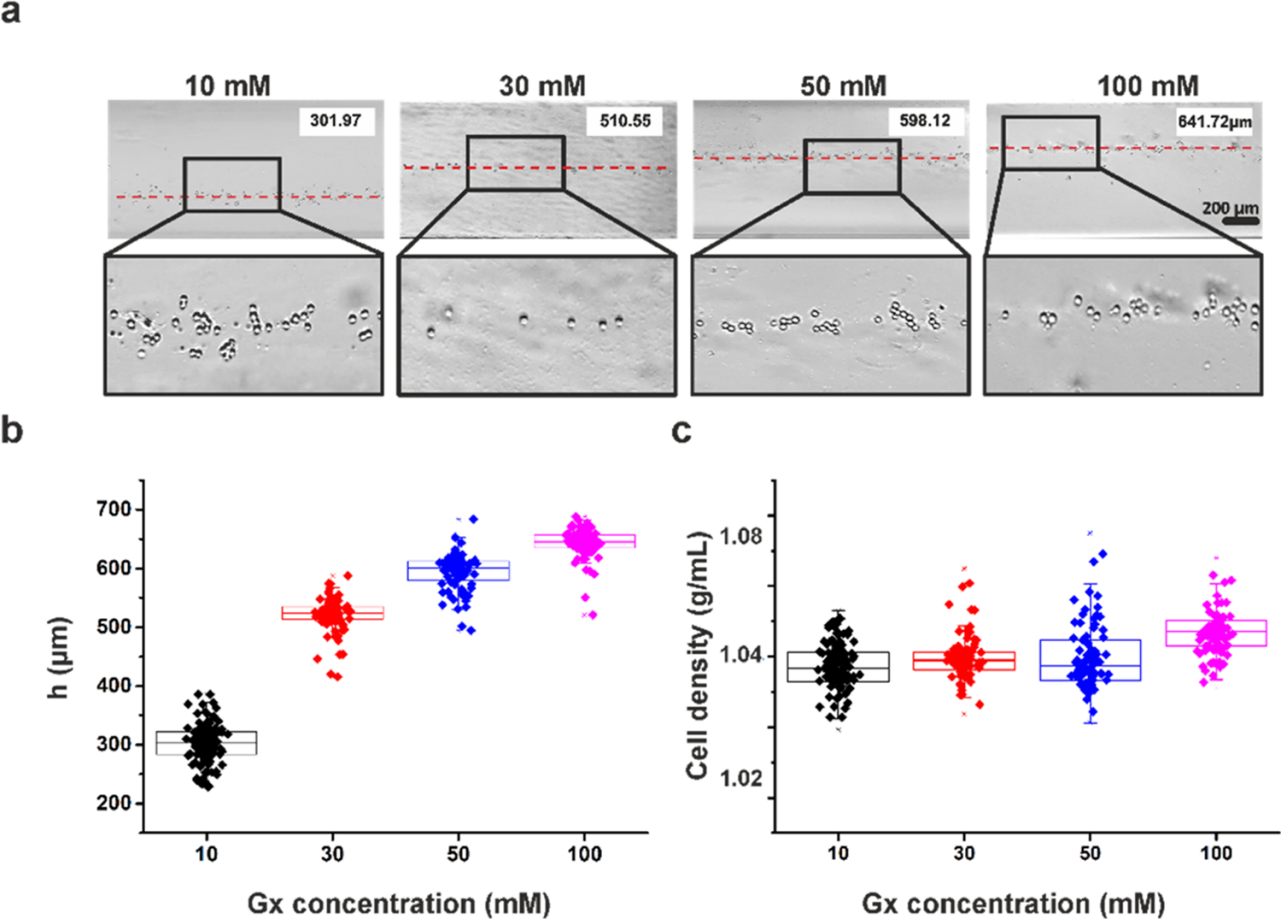
Evaluation of un-/differentiated SH-SY5Y cells at 10, 30, 50, and 100 mM Gx; (a) levitation height profiles by light microscopy, (b) levitation height (*h*) distribution, and (c) cell density distribution (*n* = 3).

The MagLev platform was tested by un-/differentiated cells with varied concentrations of Gx (10, 30, 50, and 100 mM) to find out the optimum paramagnetic agent concentration before sorting differentiated cells. For this purpose, the levitation capability of Gx was evaluated by light microscopy. Levitation height (*h*), and cell density values were measured by evaluating the light microscopy images (Figure 4). Cells reached equilibrium levitation height, where magnetic, buoyancy, and gravitational forces acting on cells were balanced within 30 min. The gradual increase in Gx concentration leads to a proportional increase in the magnetic force, causing cells to levitate at a higher position^21,22,43^ and more rapidly into the equilibrium levitation height.^21^ While the Gx concentration increased from 10 to 100 mM, the levitation height changed from 301.97 to 641.72 μm, as shown in Figure 4a,b. On the other hand, the calculated cell density remained in a similar range across different Gx concentrations, as expected (Figure 4b,c). The sorting of un-/differentiated cells was investigated with 30 mM Gx, as 10 mM Gx was not able to effectively levitate PSMs with a density above 1.06 g/mL (Supporting Figure 2), as well as cells at a certain levitation height, which leads to a random distribution of cells (Figure 4). On the other hand, higher Gx concentrations, such as 100 mM, were not used for sorting of cells since increasing Gx concentration leads to decrease in cell viability as shown in Figure 3.^43^

**Figure 5:**
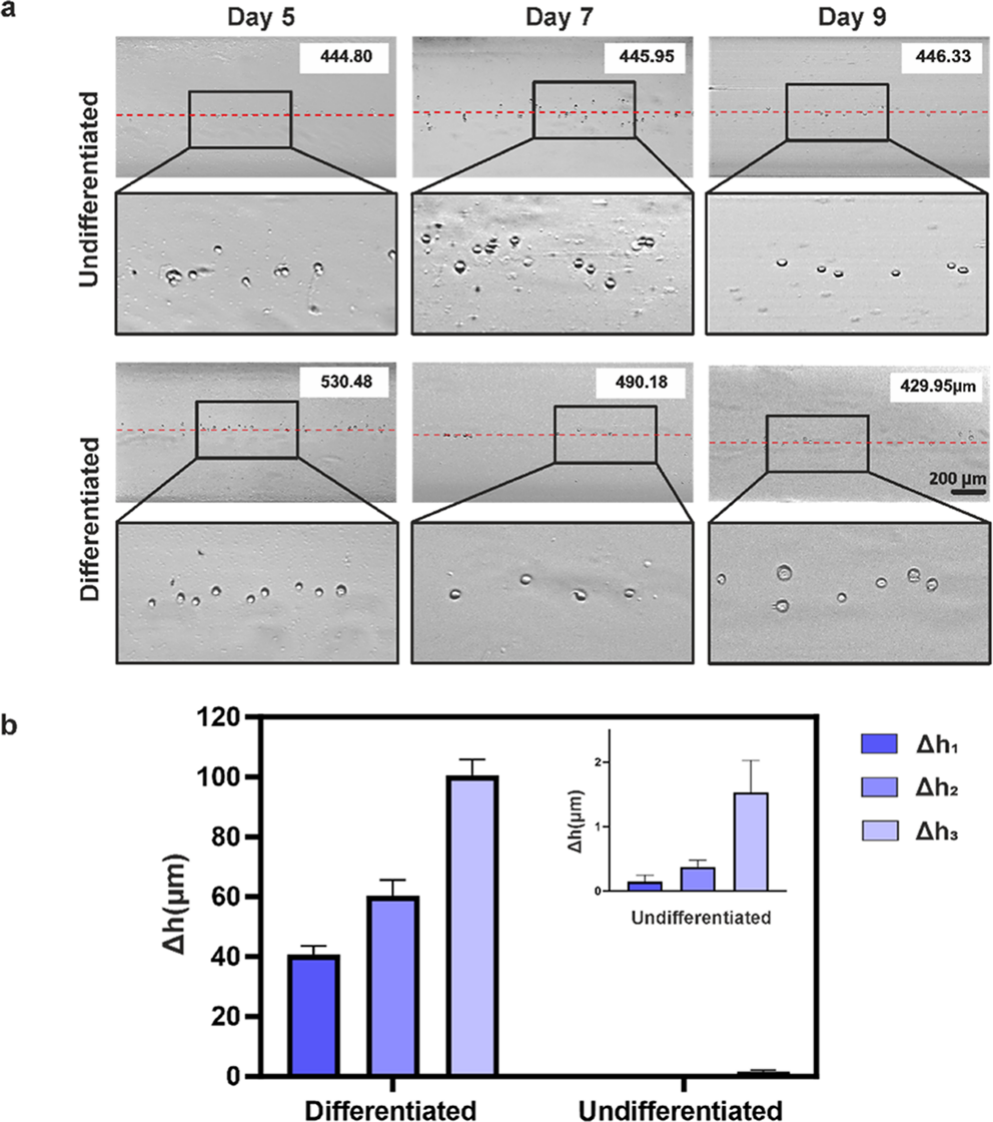
(a) Light microscopy images of levitation height profiles of un-/differentiated SH-SY5Y cells on days 5, 7, and 9 in 30 mM Gx (scale bar: 200 μm). (b) Levitation height differences (Δ*h*) of un-/differentiated cells; Δ*h*_1_ (day 5–day 7), Δ*h*_2_ (day 7–day 9), and Δ*h*_3_ (day 5–day 9).

Next, neuronal differentiation of SH-SY5Y cells at a single-cell level was characterized by using 30 mM Gx via MagLev (Figure 5). Neuronal differentiation results in increased cell mass due to the higher expression levels of neuronal markers such as MAP2, NSE, Synaptophysin, β-III tubulin, and NeuN,^10,17,42^ which leads to an increase in cell density. Since this increase will change the balance of the forces acting on the cells (magnetic, gravity, and buoyancy), it is expected that the levitation height of the cells will decrease.^21,43^Figure 5a shows the levitation height profiles of cells on days 5, 7, and 9. Each levitation height corresponds to cells harvested on different days of the differentiation process (day 5, day 7, and day 9). Consequently, each image presented in Figure 5a represents cells belonging to the same population, resulting in the alignment of cells in similar positions. The levitation height of un-/differentiated cells remained significantly stable over time as there was no change in cell density. Compared to un-/differentiated cells, the levitation height of differentiated cells was higher on day 5 and day 7, and also they reached the lowest levitation height of 429.95 μm on day 9. This decrease in levitation heights can be attributed to the increasing cell density due to higher expression levels of neuronal markers following neuronal differentiation.^42^Figure 5b illustrates the variation in levitation height difference (Δ*h*) of both un-/differentiated and differentiated cells over time. Un-/differentiated cells showed insignificant differences in Δ*h*: Δ*h*_1_ (day 5–day 7), Δ*h*_2_ (day 7–day 9), and Δ*h*_3_ (day 5–day 9), which is 0.15, 0.38, and 1.53 μm, respectively. As expected, there is a proportional increase between the levels of differentiation from day 5 to day 9. While Δ*h*_1_ exhibited the lowest value of 40.62 μm, Δ*h*_2_ was obtained as 60.23 μm. The most significant difference was obtained between day 5 and day 9 at 100.53 μm (Δ*h*_3_), representing the highest level of differentiation. In the literature, a few reports showing how cell density affects differentiation exist. It has been shown that neuronal cell density varies based on the differentiation status. During cortical development, cells from embryonic rat cerebral cortex were separated according to their buoyant density based on cell differentiation and proliferation status using Percoll gradients.^32^ Our findings are also consistent with the literature showing that an increase in cell mass occurs, which can be attributed to the increased expression of intracellular proteins after differentiation.^42^ These studies demonstrated that the differentiation process can result in cell density change, serving as a potential indicator for the detection of neuronal differentiation.

**Figure 6:**
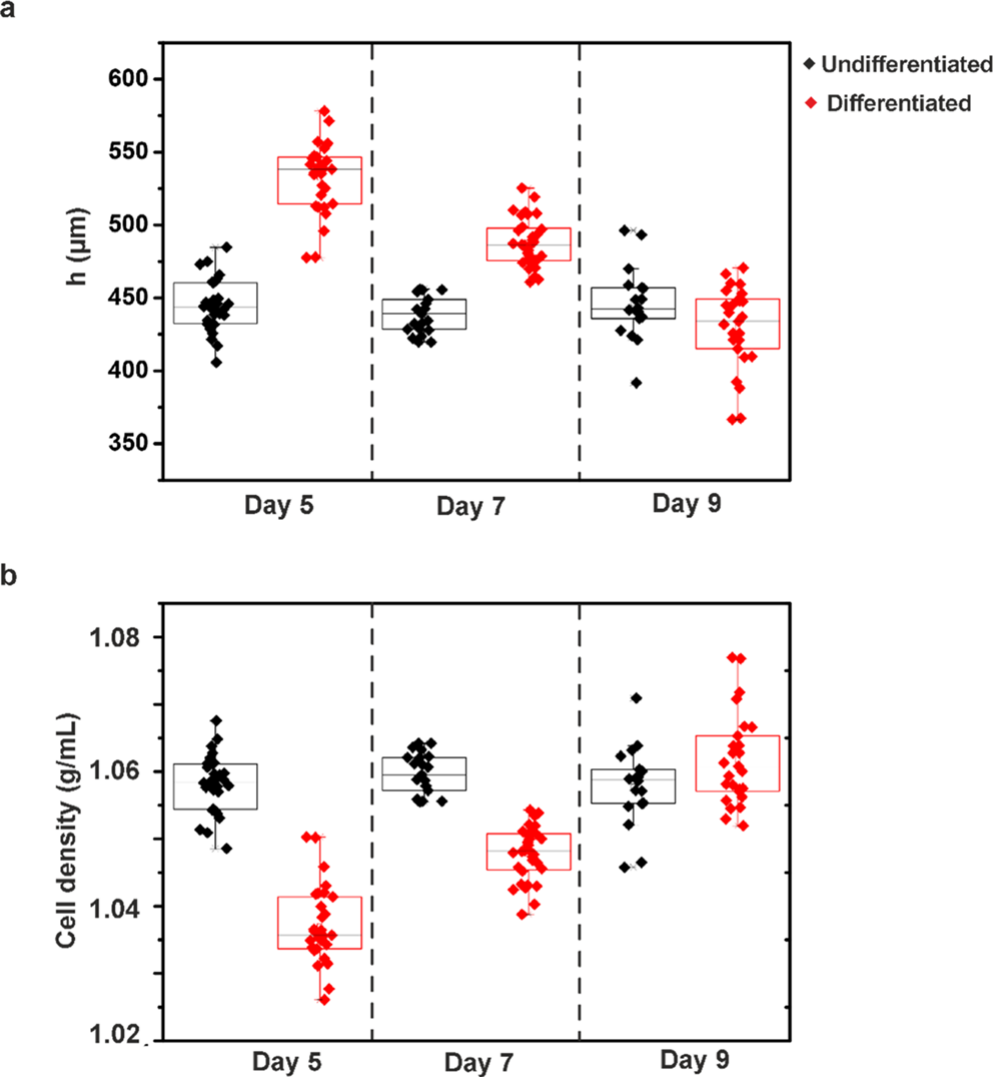
Comparison of un-/differentiated cells based on (a) levitation height (*h*) and (b) cell density distribution on days 5, 7, and 9 (*n* = 3, all data distributed normally, *p* < 0.05).

Figure 6 demonstrates levitation height (Figure 6a) and cell density distribution (Figure 6b) of un-/differentiated SH-SY5Y. The density values of the un-/differentiated cells were calculated using a density-based calibration curve. While the levitation height of un-/differentiated cells did not change over days, which remained around 445 μm, differentiated ones decreased from 530.48 to 429.95 μm (Figure 6a). The density of differentiated cells was found to be lower than that of un-/differentiated cells on days 5 and 7, and the difference between both groups decreased on day 9 (Figure 6b). Un-/differentiated cell density did not change significantly, which remained at 1.05 g/mL, while differentiated cell density increased from 1.04 to 1.06 g/mL from days 5 to 9, which is also correlated with Figure 6. In addition, the density of the differentiated cells with RA on day 5 was lower compared to the RA-BDNF treated group. This can be explained by the difference between RA and RA-BDNF treated groups in terms of volume changes. This difference can be attributed to the larger volume of cells treated with RA alone, whereas the RA-BDNF treated group has a smaller cell body, as outlined by previous reports.^45^ The levitation height and density of both un-/differentiated cells have exhibited normal distribution (*p* > 0.05) on samples of each day. A significant difference was observed in the density of differentiated groups between day 5–day 9 and day 7–day 9 (*p* < 0.05), while un-/differentiated groups exhibited no significant difference (ns) between samples of each day using one-way analysis of variance (ANOVA) followed by Tukey test. As a result, these findings suggest that MagLev technique could contribute to the investigation of neuronal differentiation by evaluating cell density.

## Conclusions

This study demonstrated the sorting of SH-SY5Y cells by the MagLev technique to analyze neuronal differentiation based on cell density difference. Here, prior to sorting, differentiation of SH-SY5Y was conducted with the sequential treatment of RA and BDNF. Neuronal differentiation was monitored through neurite length extension, and the highest neurite length was observed on day 9, which is 151.69 μm. The neuronal marker expression of un-/differentiated cells was investigated by immunostaining of β-III tubulin and NeuN. On day 9, differentiated cells showed 2- and 5-fold higher β-III tubulin and NeuN expression, respectively, compared to un-/differentiated cells. Prior to the sorting of cells, Gx cytotoxicity was assessed using live/dead and MTT assays, and the optimum Gx concentration was found to be 30 mM, which was able to levitate cells without any random distribution. Later, cells were sorted using the MagLev platform on days 5, 7, and 9. While the differentiated cells reached the lowest levitation height, which is 429.95 μm on day 9, the un-/differentiated cells remained stable over time. Moreover, the un-/differentiated cells displayed minimal variations in cell density, which remained at 1.05 g/mL through 9 days, whereas the density of differentiated cells increased from 1.04 to 1.06 g/mL due to higher expression of the neuronal markers during differentiation. These findings indicate that differences in cell density can be used as distinctive properties for sorting SH-SY5Y cells via MagLev. This supports the idea that cellular differentiation can result in notable biochemical and morphological changes, with cell density emerging as a property influenced by this process. This method proved to be time-efficient, label-free, easy to use, and cost-effective to sort cells, which mitigated conventional techniques’ drawbacks. Besides, the MagLev method has the potential to be a reliable tool for evaluating cell differentiation, extending beyond its application to SH-SY5Y cells. It can be adapted to broader fields to investigate various biological processes, ranging from neuroscience, where the differentiation of neuronal cells is of particular interest, to regenerative medicine, where understanding cellular behavior is crucial for therapeutic applications.

## Materials and Methods

### Standard Cell Culture and Differentiation of SH-SY5Y Cells

SH-SY5Y (human bone-marrow neuroblastoma, ATCC CRL-2266) cell line was cultured in high-glucose DMEM (GIBCO, Thermo Fisher Scientific) containing l-glutamine and supplemented with 15% fetal bovine serum (GIBCO, Thermo Fisher Scientific) and 1% penicillin/streptomycin (GIBCO, Thermo Fisher Scientific). The cells were cultured up to ∼90% confluency in a humidified environment (5% CO_2_, 37 °C). The harvested cells were used for differentiation and for further studies.

Retinoic acid (RA, Acros organics) and brain-derived neurotrophic factor (BDNF, ABclonal) were used for differentiation of the SH-SY5Y cell line. Differentiation was induced by 10 μM RA (in DMSO) with 1% FBS on day 1 and cell culture was maintained for 5 days, and the medium was refreshed every other day. On day 5, 50 ng/mL BDNF was supplemented into the cell medium; again, the medium was refreshed every other day until day 9. Cellular morphology and differentiation progress were monitored by Zeiss Axio Observer microscopy.

The cytotoxicity of the paramagnetic agent on SH-SY5Y was investigated by live/dead and MTT analyses. For this purpose, 1 × 10^4^ cells were seeded to 96-well plates with 10, 30, 50, and 100 mM Gadobutrol (Gx; Gadovist, Bayer) and screened up to day 7. CytoCalcein Green and propidium iodide (PI) dyes (AAT Bioquest) were used for live/dead analysis and visualized by a fluorescence microscope. MTT analysis was carried out using a Multiskan GO Microplate Spectrophotometer (Thermo Fisher Scientific).^43^

### Characterization of Neuronal Differentiation

Neuron J (ImageJ software, NIH) is a program that traces and quantifies neurites. The neurite length of cells exposed to RA and BDNF was measured on days 0, 5, 7, and 9 by Neuron J,^44^ and at least three individual replicates were used.

Immunostaining assay was performed to examine and confirm the expression of β-III tubulin and neuronal nuclei (NeuN), which are neuronal-specific markers for differentiation.^42,44^ Un-/differentiated cells (day 5, 7, and 9) were fixed with 4% paraformaldehyde, permeabilized with 0.1% Triton X-100, and blocked with 1% bovine serum albumin (BSA). Anti-β-III tubulin (ABclonal) and anti-NeuN (ABclonal) were applied. Further, DAPI staining was done to visualize the cell nuclei. Stained cells were monitored by fluorescence microscopy, and images were used to calculate the fluorescence intensity by ImageJ Software (NIH).

### Cell Sorting via MagLev

Detection of un-/differentiated cells was conducted using the MagLev setup, the fabrication details of which were depicted previously.^43^ Briefly, N52-grade magnets were assembled in anti-Helmhotz configuration and mirrors were fixed into the setup at 45°. All analyses were done in the capillary channel, which was positioned between two magnets (Supporting Figure 2). MagLev platform was calibrated, as reported elsewhere;^22,43^ for this purpose, 1.02, 1.04, 1.06, 1.08, 1.09, and 1.13 g/mL density marker polystyrene microbeads (PSMs, Cospheric LLC) were utilized. They were suspended in the medium, which contains 10, 30, 50, or 100 mM Gx, and loaded into the capillary channel. Image and data analyses were performed to plot calibration curves based on their levitation height (Supporting Figure 3). In addition, single-cell density was calculated via MATLAB software 2018b using the calibration curve, as described elsewhere.^46^ Moreover, the levitation capability of Gx was tested on SH-SY5Y cells for 10, 30, 50, and 100 mM Gx. Un-/differentiated cells were harvested on days 5, 7, and 9 and introduced into the capillary channel with 30 mM Gx for sorting. The cells were aligned at equilibrium levitation height for around 30 min, and then light microscopy images were obtained for further analysis. Levitation height was determined using the obtained images, and a density calculation was carried out utilizing the calibration curve.

### Statistical Analysis

Cell viability and proliferation experiments were done from at least three independent replicates, and data were expressed as mean ± SD. One-way and two-way ANOVA followed by Tukey test for multiple comparison were performed by GraphPad Prism 9 software (GraphPad Prism, Inc., San Diego). The statistical significance between groups was considered as *p ≤ 0.05, **p ≤ 0.01, ***p ≤ 0.001, ****p ≤ 0.0001.

## Supporting Information Available

MagLev setup components and view; standard calibration curve of MagLev platform; differentiation of SH-SY5Y cells only with RA for 9 days (PDF)
