# Supplementary material for: Exploring Neuronal Differentiation Profiles in SH-SY5Y Cells through Magnetic Levitation Analysis
Source: ACS Omega. 2024 Mar 22;9(13):14955–62. doi: 10.1021/acsomega.3c08962 (PMC10993277; doi:10.1021/acsomega.3c08962)
Supplement: Supplementary file 1 — ao3c08962_si_001.pdf [file ao3c08962_si_001.pdf]

Exploring Neuronal Differentiation Profiles in SH-SY5Y Cells through Magnetic Levitation Analysis

Rumeysa Bilginer Kartal[1], *Ahu Arslan Yildiz*[1*]

[1]Department of Bioengineering, Izmir Institute of Technology (IZTECH), 35430 Izmir, Turkey

*Corresponding author: e-mail: ahuarslan@iyte.edu.tr

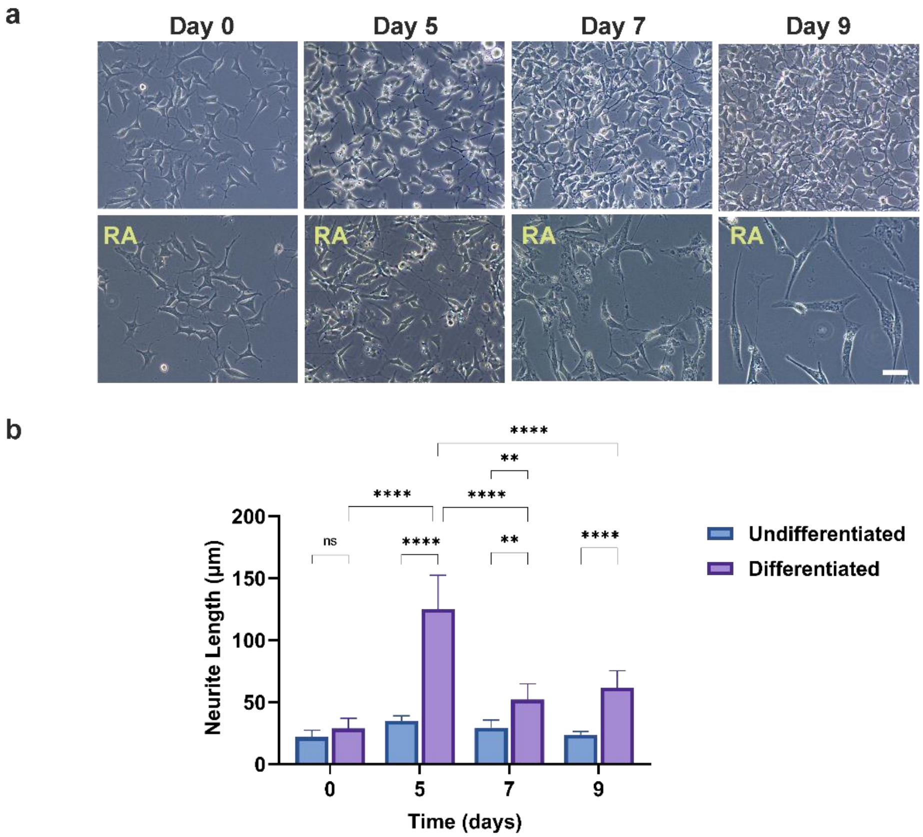

Supplementary Figure 1 a) Morphology and b) neurite extension analysis of un/differentiated SH-SY5Y after treatment by RA for 9 days. (n:10, ns: not significant, **$p < 0.01$ ****$p < .0001$). *Scale bar: 50 µm*

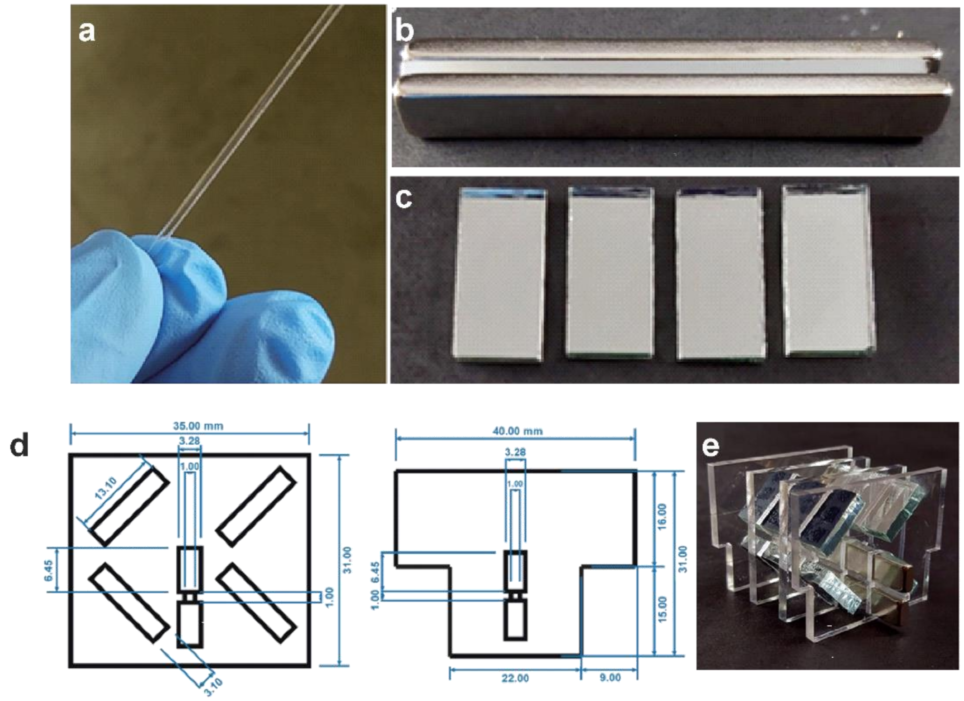

Supplementary Figure 2 Components and view of MagLev setup a) Capillary channel b) N52 Magnets c) Mirrors d)Vectorial drawing and e) 3D view of MagLev setup

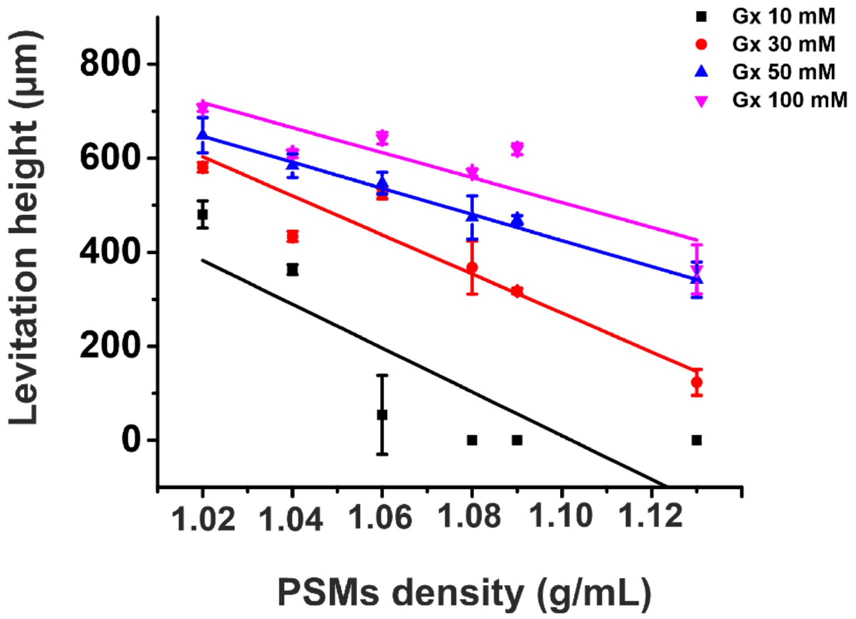

Supplementary Figure 3 Calibration of MagLev platform with PSMs beads and Gx
